# Quantification of Global DNA Methylation in Canine Melanotic and Amelanotic Oral Mucosal Melanomas and Peripheral Blood Leukocytes From the Same Patients With OMM: First Study

**DOI:** 10.3389/fvets.2021.680181

**Published:** 2021-08-24

**Authors:** Nayra Villar Scattone, Tatiane Moreno Ferrarias Epiphanio, Karine Germano Caddrobi, Juliana Shimara Pires Ferrão, Francisco Javier Hernandez-Blazquez, Ana Paula de Melo Loureiro, Cristina de Oliveira Massoco, Maria Lucia Zaidan Dagli

**Affiliations:** ^1^Laboratory of Experimental and Comparative Oncology, School of Veterinary Medicine and Animal Science, University of São Paulo, São Paulo, Brazil; ^2^PROVET Veterinary Hospital, São Paulo, Brazil; ^3^Department of Biomedicine, Faculty Anhanguera, Guarulhos, Brazil; ^4^Department of Surgery, School of Veterinary Medicine and Animal Science, University of São Paulo, São Paulo, Brazil; ^5^Department of Clinical and Toxicological Analysis, School of Pharmaceutical Sciences, University of São Paulo, São Paulo, Brazil; ^6^Laboratory of Pharmacology and Toxicology, School of Veterinary Medicine and Animal Science, University of São Paulo, São Paulo, Brazil

**Keywords:** cancer, DNA methylation, melanoma, dog, cell proliferation

## Abstract

Oral mucosal melanomas (OMMs) are aggressive and resistant cancers of high importance in veterinary oncology. Amelanotic OMM produces comparatively less melanin and is considered to be more aggressive than melanotic OMM. Global DNA methylation profiles with hypomethylated or hypermethylated patterns have both been associated with aggressive neoplasms; however, global DNA hypomethylation seems to correlate to higher aggressiveness. Accordingly, global DNA methylation in peripheral blood leukocytes has been investigated to understand the role of systemic or environmental factors in cancer development. This study aimed to quantify global DNA methylation in canine melanotic and amelanotic OMM samples and in the peripheral blood leukocytes of the same dogs. Tumor tissue samples were collected from 38 dogs, of which 19 were melanotic and 19 were amelanotic OMM. These were submitted to immunohistochemistry (IHC) with anti-5-methylcytosine (5mC) and anti-Ki67 primary antibodies. Ki67- and 5mC-positive nuclei were manually scored with the help of an image analysis system. Peripheral blood samples were collected from 18 among the 38 OMM-bearing dogs and from 7 additional healthy control dogs. Peripheral blood leukocytes were isolated from the 25 dogs, and DNA was extracted and analyzed by high-performance liquid chromatography (HPLC) for global DNA methylation. The pattern of global DNA methylation in both canine melanotic and amelanotic OMM indicated higher percentages of weakly or negatively stained nuclei in most of the OMM cells, presuming predominant global DNA hypomethylation. In addition, Ki67 counts in amelanotic OMM were significantly higher than those in melanotic OMM (*p* < 0.001). Global DNA methylation different immunostaining patterns (strong, weak or negative) correlated with Ki67 scores. Global DNA methylation in circulating leukocytes did not differ between the 9 melanotic and 9 amelanotic OMM or between the 18 OMM-bearing dogs and the 7 healthy dogs. This study provides new information on canine melanotic and amelanotic OMM based on global DNA methylation and cell proliferation.

## Introduction

Melanoma is the most common malignant tumor in the oral cavity of dogs, accounting for 30–40% of all oral cavity cancers in dogs and 7% of all types of cancer in this species (1, 2). Breeds with higher mucosal pigmentation have a higher risk of developing oral melanoma, particularly the Scottish Terrier, Golden Retriever, Dachshund, Cocker Spaniels, and Poodles. An epidemiological study involving 2,350 affected dogs revealed that poodles are at a high risk of developing oral melanomas (3). Melanomas generally occur in middle-aged to older dogs, with average age of 9 years, regardless of gender. In dogs, 40% of OMM cases without lymphadenomegaly already have lymph node metastasis. In addition, the presence of metastatic lymph nodes at the time of diagnosis accounts for ~25% (2, 4). Goldschmidt et al. (5) reported that melanomas in canines could be classified in melanotic (or melanocytic), when they kept the capacity to produce melanin, and amelanotic (or amelanocytic) when this capacity was totally or partially lost.

Melanotic and amelanotic OMMs are considered to be aggressive cancers in dogs. A first study from our group revealed, for the first time, differences between melanotic and amelanotic OMM, so that amelanotic OMM had more mitotic cells than melanotic counterparts, differed in the expression of connexins (i.e., the gap junction proteins) and dogs bearing amelanotic OMM presented a shorter lifespan in comparison to those with melanotic OMM (6).

Canine OMM can be considered a good model for human OMM (2, 3, 7–9) as both harbor NRAS mutations. However, according to Brocca et al. (10), single nucleotide polymorphysms are not a frequent event in *KIT* activation in canine OMM, and these differ from human OMM in this aspect.

In a study involving 95 dogs, canine cDNA sequencing data for six genes relevant to human melanoma classification detected somatic mutations in the NRAS and PTEN genes at the human hotspot sites, except in BRAF in oral melanomas (3). Wong et al. (11) performed a cross-species analysis by sequencing tumor-germline pairs from 46 primary human muscosal, 65 primary canine oral and 28 primary equine melanoma cases from mucosal sites.The data revealed recurrently mutated driver genes shared between species such as *NRAS, FAT4, PTPRJ, TP53* and *PTEN*, and pathogenic germline alleles of *BRCA1, BRCA2* and *TP53*.

Investigations into epigenetic characteristics of canine OMM have been scarce in recent years.

Cancer-associated epigenetic changes include global hypomethylation, site-specific hypomethylation and hypermethylation, and modification of chromatin linked to tumor suppressor gene silencing and oncogene activation (12). In several studies, reductions in global DNA methylation and hypermethylation of tumor suppressor genes are associated with carcinogenesis (13, 14). Although these patterns are independent, they may coexist in neoplasms and have been considered as biomarkers associated with cancer (2, 15, 16).

The study of global DNA methylation by using antibodies anti- 5mC started in the 1990's by Dr Alain Niveleau, from the Faculté de Médecine Claude Bernard Lyon I in France, who initially produced the antibody against 5mC. Piyathilake et al. (17), compared the results of global DNA methylation evaluated by radiolabeled methyl incorporation (CPM/microg of DNA) with immunohistochemical staining of the same tissue sections with a monoclonal antibody developed against 5-methylcytosine (5mC). The results suggested that both radiolabeled methyl incorporation assay and immunostaining for 5mC could be used to demonstrate hypomethylation of DNA in squamous cell carcinoma tissues compared to matched uninvolved tissues.

Based on our previous studies using anti-5-methylcytosine (5mC) immunostaining, global DNA methylation patterns was found to differ in mast cell tumor grades (18): grade 3, high-grade mast cell tumors, which present worse prognosis, showed predominant weak immunostaining for 5mC. Moreover, canine mammary cancers (19) were identified to develop a higher aggressiveness in hypomethylated (predominantly 5mC-weakly immunostained) tumors.

The evaluation of peripheral blood leukocyte global DNA methylation has been an interesting approach in epigenetics research (20, 21), as it allows us to investigate the influence of the environment on cancer development. Global hypomethylation in peripheral blood cell DNA has been associated with increased risk and prognosis of various cancers in humans (22). Alterations in DNA methylation in peripheral blood cells may not necessarily correlate with epigenetic changes in tumors; however, global DNA hypomethylation in peripheral blood leukocytes may reflect the genomic instability of an individual, which may lead to cancer development (23, 24). Recently, Epiphanio et al. (25) sought to determine whether peripheral blood global DNA methylation was associated with canine multicentric lymphomas. Based on their findings, dogs with non-Hodgkin lymphomas presented global DNA hypomethylation of circulating leukocytes compared to healthy dogs.

No reports regarding global DNA methylation in canine OMM, nor reports linking the global DNA methylation of peripheral blood leukocytes with oral melanoma in dogs, have been published in the field of veterinary medicine. Therefore, the aim of this study was to evaluate global DNA methylation by 5mC immunostaining and cellular proliferation in melanotic and amelanotic OMM, and quantify global DNA methylation in the peripheral leukocytes of dogs with OMM.

## Materials and Methods

### Ethics and OMM Samples

The study protocol was approved by the Committee of Ethics on the Use of Animals (CEUA) of the School of Veterinary Medicine and Animal Science (Process number: 9522230517) and the School of Medicine of the University of São Paulo (Process number: 997/18). All dog owners signed informed consent forms.

For this study, OMM samples were acquired from two sources.

The first *set of 18 OMM* was obtained from dogs enrolled at the Provet Veterinary Hospital and Pet Life Care Hospital in São Paulo. Amelanotic and melanotic OMM samples (9 samples of each) were obtained from the animals during the surgical procedures for removal of tumors, or electrochemotherapy procedures, and blood samples from the same dogs were obtained for the quantification of leukocyte global DNA methylation. Blood was also collected from seven healthy dogs with similar ages to serve as the control. The groups were composed of male and female dogs, with or without defined breeds. The exclusion criteria were: history of other neoplasms, use of corticosteroids and chemotherapy before sample collection, and other concomitant serious diseases. The second *set of OMM* was composed of 20 melanotic and amelanotic OMM samples (10 samples of each) in paraffin blocks, that were kindly provided by Dr Cristina de Oliveira Massoco, from the Laboratory of Pharmacology and Toxicology at the SVMAS – USP.

All OMM samples were fixed in 10% formalin and routinelly embedded in paraffin wax. The 5μm sections were stained with Hematoxylin and Eosin to confirm the diagnosis before being submitted to immunohistochemistry. All amelanotic OMM had <50% pigmented cells (26), and their diagnosis was confirmed by immunostaining with the anti-Melan A antibody (27).

### Blood Sampling for the Analysis of Leukocyte Global DNA Methylation

Four mL of peripheral blood, obtained via venipuncture, was collected from 18 melanoma-bearing and the 7 control dogs in EDTA tubes. Blood was centrifuged at 3,000 × g for 10 min at 4 °C, and the buffy coat containing leukocytes was separated. Samples were stored at −80 °C for subsequent DNA extraction.

### Immunohistochemistry

All melanoma samples were obtained immediately after surgery, fixed in 10% formalin, and embedded in paraffin wax. The obtained 5 μm sections were subjected to immunohistochemistry with the following antibodies: anti-Melan A, anti-5-methyl cytosine (5mC), and anti-Ki-67 (Table 1). Additional slides representing samples from the negative control were subjected to the same immunohistochemical protocol, except the treatment with the primary antibodies.

**Table 1:** Primary antibodies used for immunohistochemistry and their dilutions.

**Antibody**	**Species**	**Clone**	**Dilution**	**Antigen recovery**
Anti-MelanA (Dako®)	Mouse (monoclonal)	A103	1:50	Citrate buffer
Anti-Ki67 (Dako®)	Mouse (monoclonal)	MIB-1	1:50	Citrate buffer
Anti-5MeCyt (Abcam®)	Mouse (monoclonal)	33D3	1:100	Citrate buffer

After deparaffinization, the slides were gradually hydrated in ethanol, and the peroxidase was blocked for 40 min at 37°C in a solution containing hydrogen peroxide. The cells were then washed with phosphate buffer (PBS) and subjected to antigenic recovery in a Pascal pressure cooker (DAKO®) for 15 min with citrate buffer (pH 6.0). Thereafter, the slides were washed with PBS and incubated overnight at 4°C with specific primary antibodies.

Following overnight incubation, the slides were washed with PBS and incubated with Polymer EnVision ™ + Dual Link System-HRP® polymer for 30 min in the oven, according to the manufacturer's recommendations. After washing with PBS, the reactions were revealed with 3-amino-9-ethylcarbazole (AEC) for melanotic OMM or diaminobenzidine (DAB) for amelanotic OMM (DAKO®). The slides were counterstained with hematoxylin, washed with 0.5% ammoniacal water, washed under running water, subjected to diaphanization (protocol of ethyl alcohol and xylol), and finally assembled. The bleaching of melanotic melanomas was not performed in this study as bleaching modifies the immunogenicity of the tissue antigens, which may interfere with the final results of immunostaining.

Immunostaining with anti-5mC and anti-Ki67 was performed in separated batteries, with all samples from the same set placed in each battery to enable uniformity of the exposure time and quantification of the staining intensity.

### Quantification and Scoring of 5mC- and Ki67-Positive Nuclei

An optical microscope (NIKON, Tokyo, Japan) linked to the Image-Pro Plus system (v.4.5.0.29; Media Cybernetics, Rockville, MD, USA) was used to quantify the 5mC- or Ki67-positive nuclei in the canine OMM. Digital photomicrographs were captured under the same light conditions, and 5 to 8 microscopic fields (40 × objective) of each sample were analyzed (counting and classifying at least 500 melanoma cells per sample) by the author NVS.

OMM cells were considered positive for 5mC if they presented nuclei with brown/gold (diaminobenzidine, DAB) or red (AEC) coloration. Nuclear staining intensity was classified as 0 (no staining), 1+ (weak staining), or 2+ (strong staining). The percentage of positive (2+, 1+) or negative nuclei was obtained for each photomicrograph, and then the percentage of 5mC 2+, 1+ or 0 was obtained for each tumor. The mean (for parametric tests) or median (for non-parametric tests) of the values for melanotic or amelanotic OMM was then obtained, and used for the statistical comparisons.

To quantify Ki67-positive cells, nuclei were scored as positive or negative on the Ki67 immunostained slides. At least 500 cells in 5 to 10 high-power fields (40 × objective) were counted per slide. The percentage of positive cells in each slide was calculated, and then a mean was obtained with the sum of the percentage of positive nuclei in each group (amelanotic or melanotic OMM), divided by 19.

### DNA Extraction and High Performance Liquid Chromatography for Quantifying Global DNA Methylation in Peripheral Blood Leukocytes

DNA extraction and purification were carried out according to the protocol provided in the Gentra Puregene® DNA extraction kit (QIAGEN Sciences, Maryland, USA). Briefly, the previously collected blood leukocyte fractions from 25 dogs (3 groups, 25 samples) were added to 600 μL of erythrocyte lysis solution (*RBC Lysis Solution*, Gentra Puregene® kit) after 10 min of incubation at 25°C room temperature. After the samples were centrifuged at 2,000 × g for 5 min at 4°C, the supernatant was carefully discarded. The samples were then added to 500 μL of cell lysis solution (*Cell Lysis Solution*, Gentra Puregene® kit) and 25 μL of ribonuclease A (15 mg/mL). Samples were subsequently incubated at 37°C for 15 min, and the protein was precipitated by the addition of 300 μL of protein precipitation solution (*Protein Precipitation Solution*, Gentra Puregene® kit) and centrifuged at 3,000 × g for 10 min at 4°C. The supernatant was transferred to a tube containing 1 mL of cold 2-isopropanol, and the precipitated DNA was collected via centrifugation at 2,000 × g for 5 min at 4°C. The DNA was washed with 1 mL of 70% ethanol, dried, and resuspended in 20 μL of 0.1 mM deferoxamine solution. The DNA concentration was determined by measuring the UV absorption at λmax: 260 nm (Libra S12 Spectrophotometer, Biochrom, Cambridge, UK), and DNA purity was assessed using the UV absorbance ratio at λmax: 260/λmax: 280 nm. Aliquots of 10-μg DNA samples in 0.1 mM deferoxamine solution were added to 2.5 μL of Tris-HCl/MgCl_2_ buffer (200 mM, pH 7.4) and 1 unit of DNase I. Samples were incubated at 37°C for 60 min. After the addition of phosphodiesterase I (0.001 units) and alkaline phosphatase (1.2 unit), incubation was continued for another 60 min at 37°C. At the end of incubation, the samples were centrifuged at 9,300 × g for 10 min. Aliquots (10 μL) were injected into the HPLC-UV analytical system (*Shimadzu Corporation*®, Kyoto, Japan) to quantify global DNA methylation, expressed as percent 5-methyl-2'-deoxycytidine (5MeCyt), using the following equation:

$$\begin{array}{l} 5mC\;\left(\%\right)=\frac{5mC\times 100}{5mC+{dC*}_{\left(nmol\right)}} \end{array}$$

^*^deoxycytidine (dC).

The chromatographic condition consisted of a 250 × 4.6 mm i.d., 5 μm, Shim-pack VP-ODS column (Shimadzu, Kyoto, Japan) with a C18 4.0 × 3.0 mm pre-column (Phenomenex, Torrance, CA) eluted with a gradient of 0.1% formic acid in water (solution A) and methanol:water (1:1) with 0.1% formic acid (solution B) at a flow rate of 1 mL/min and 35°C. The following conditions were employed: from 0 to 25 min, 0 to 40% B; from 25 to 26 min, 40 to 0% B; and from 26 to 40 min, 0% B. The PDA detector was set at 286 nm. Calibration curves were constructed at intervals of 0.01 to 1.2 nmol of 2′-deoxycytidine (dC) and 0.002–0.04 nmol of 5mC.

### Statistical Analysis

GraphPad Prism® software was used for the statistical analyses. After the normality test, 5mC- or Ki67- positive nuclei quantifications were compared between melanotic and amelanotic OMM. The Kruskal Wallis non-parametric test, with the Dunn's *post-test*, was used to compare 5mC-positive cell medians between melanotic and amelanotic OMM (considering *p* < 0.05). The Student's *t*-test was employed to compare the percentages of Ki67-positive nuclei between melanotic and amelanotic OMM; *p* < 0.05 was set as the significance limit. The Student's *t*-test and ANOVA were employed as the statistical tests to quantify the global DNA methylation in blood leukocyte. The Spearman's rank correlation coefficient was used to define the level of correlation between 5mC and Ki67 scores.

## Results

### Subjects Characteristics

In this study, samples from 19 dogs with melanotic OMM and 19 with amelanotic OMM (ages: 8–16 years-old; sex ratio: 52.64% females and 47.36% males) were included. The dog breeds with melanotic OMM were Mongrels (8/19), Poodles (4/19), Beagles (1/19), Lhasa Apsos (1/19), Golden Retriever (2/19), Schnauzer (2/19) and Yorkshier (1/19), while those with amelanotic OMM were Mongrels (11/19), and Yorkshires (2/19), Pischer (1/19), Pug (1/19), Teckel (2/18), Cocker Spaniel (1/18). The control group was composed of seven healthy dogs (age: 10–14 years-old, sex: 57.14% males and 42.85% females), where 5/7 and 2/7 were Mongrels and German shepherd dogs, respectively. All OMM bearing dogs were taken to the veterinary hospital to undertake electrochemotherapy, surgery and or dendritic cell vaccination. Most dogs were in WHO stage II (4), and none of them had metastasis at the moment of diagnosis and therapeutic procedures. The characteristics of the dogs enrolled in the study are shown in Table 2.

**Table 2:** Characteristics of the dogs included in the study.

**Canine patient**	**Gender**	**Age (yrs)**	**Breed**	**WHO stage**
**Melanotic OMM**				
M1	M	15	Mongrel	II
M2	F	14	Mongrel	II
M3	M	14	Poodle	II
M4	F	14	Beagle	II
M5	F	12	Poodle	II
M6	F	16	Mongrel	II
M7	M	12	Mongrel	II
M8	M	13	Lhasa apso	II
M9	M	12	Mongrel	II
M10	M	9	Golden retriever	III
M11	F	12	Yorkshire	II
M12	F	11	Schnauzer	II
M13	M	10	Poodle	III
M14	F	15	Mongrel	III
M15	M	13	Mongrel	III
M16	M	15	Poodle	III
M17	M	13	Mongrel	II
M18	M	12	Golden retriever	III
M19	M	11	Schnauzer	II
**Amelanotic OMM**				
A1	F	11	Mongrel	II
A2	F	12	Mongrel	II
A3	F	13	Mongrel	II
A4	F	12	Yorkshire	II
A5	F	12	Mongrel	II
A6	F	14	Yorkshire	II
A7	F	14	Mongrel	II
A8	F	12	Mongrel	II
A9	M	13	Mongrel	II
A10	M	13	Pinscher	II
A11	F	16	Mongrel	III
A12	F	12	Pug	III
A13	M	8	Teckel	I
A14	M	14	Mongrel	I
A15	F	14	Cocker Spaniel	II
A16	M	15	Teckel	III
A17	F	16	Mongrel	III
A18	M	9	Teckel	III
A19	F	8	Mongrel	III
**Clinically Healthy (controls)**				
C 1	F	12	Mongrel	Clinically Healthy
C 2	M	11	Mongrel	Clinically Healthy
C3	F	10	German Shepherd	Clinically Healthy
C4	M	12	Mongrel	Clinically Healthy
C5	M	10	German Shepherd	Clinically Healthy
C6	M	14	Mongrel	Clinically Healthy
C7	F	14	Mongrel	Clinically Healthy

### Scoring of 5mC and Ki67-Positive Nuclei

Melanotic and amelanotic OMM samples were analyzed to derive their immunoreactivity to 5mC to investigate global DNA methylation.

**Figure 1:**
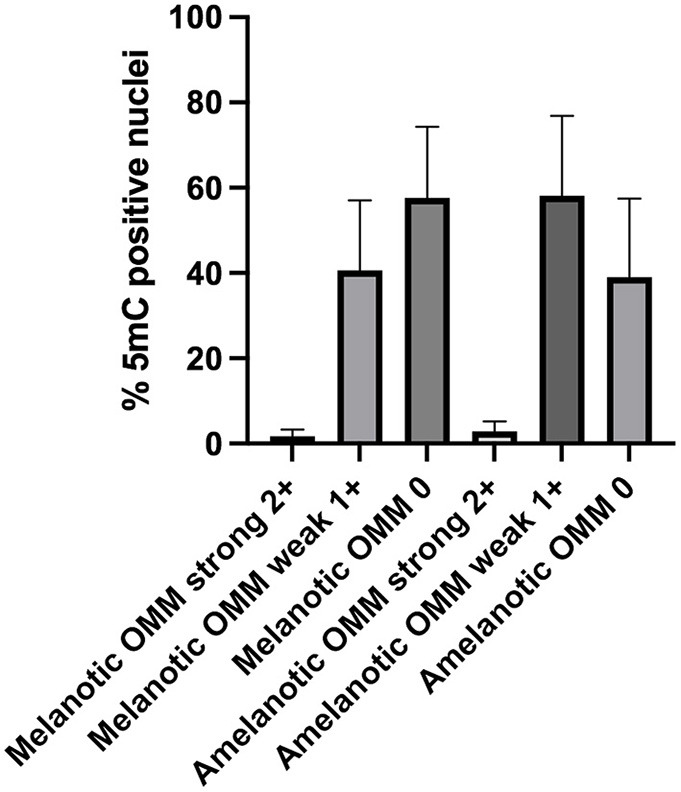
Scores of the 5mC-positive nuclei in samples of canine melanotic and amelanotic OMMs. The 5mC-positive cells were divided into strong (2+), weak (1+), or negative (0) staining. Medians were compared by Kruskal Wallis, with the Dunn's *post-test*. No significant differences were obtained. However, more than 90% of the cells in both melanotic and amelanotic OMM samples were either weakly or negatively stained for 5mC.

Nuclear immunostaining for 5mC was classified as strong (2+), weak (1+), or negative (0). Most cells from both melanotic and amelanotic OMM samples presented weak or negative staining for 5mC (see Table 3). In addition, no differences between melanotic and amelanotic OMMs regarding the strong, weak, and negative nuclei percentages was observed, as shown in Table 3 and Figure 1.

**Table 3:** Summary data of the Ki67 and 5mC quantifications in melanotic and amelanotic OMM.

	**Ki67**	**5mC 2+**	**5mC 1+**	**5mC -negative**
Melanotic OMM (*n =* 19)	21.12 ± 9.03	1.71 ± 1.57	40.64 ± 16.41	57.65 ± 16.61
Amelanotic OMM (*n =* 19)	57.32 ± 12.04^*^	2.83 ± 2.37	58.15 ± 18.76	39.02 ± 18.41

*Data represent the mean of the percentages (mean ± standard deviation)*.

* *Statistically significant, Student t-test (p < 0.001) so that amelanotic OMM have significantly higher positive nuclei*.

*Kruskal Wallis and Dunn's test (p > 0.05) were used to compare the 5mC scores for strong, weak, and negative nuclei immunoreactivity between melanotic and amelanotic OMMs*.

**Figure 2:**
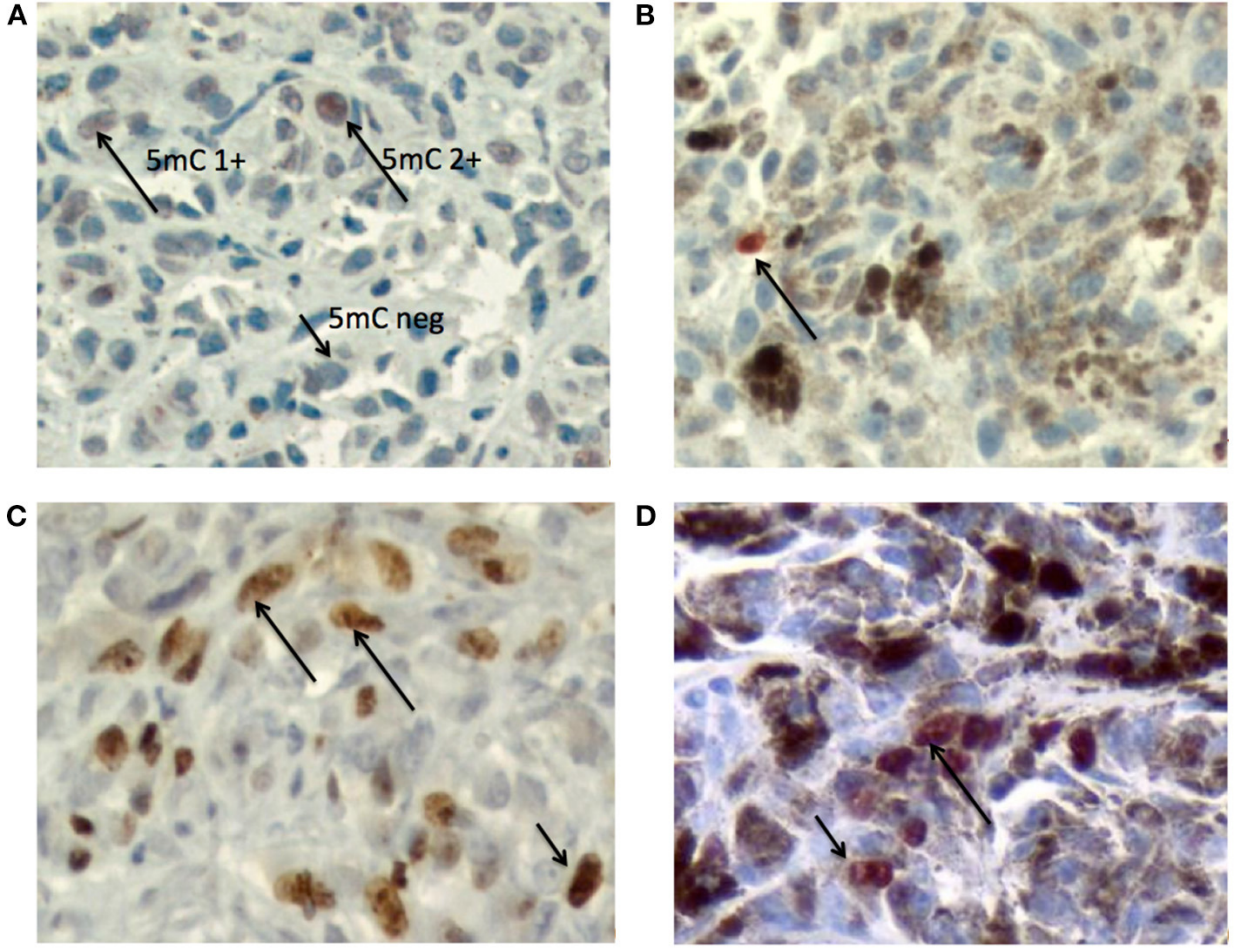
Photomicrographs of the immunostainings for 5mC and Ki67 in melanotic and amelanotic OMM. **(A)** Immunostaining of 5mC in amelanotic OMM. Arrows indicate the patterns 5mC2+ and 5mC 1+ and 5mC 0 (DAB + hematoxylin). **(B)** Immunostaining of 5mC in melanotic OMM. Arrow indicates the 5mC 2+ nucleus (AEC + hematoxylin). **(C)** Immunostaining of Ki67 in amelanotic OMM. Arrows indicate a positive nuclei (DAB + hematoxylin). **(D)** Immunostaining of Ki67 in melanotic OMM. Arrows indicate positive nuclei (AEC + hematoxylin). Objective = 40x.

Figure 2 presents example photomicrographs of the immunostainings for 5mC and Ki67 in melanotic and amelanotic OMM.

**Figure 3:**
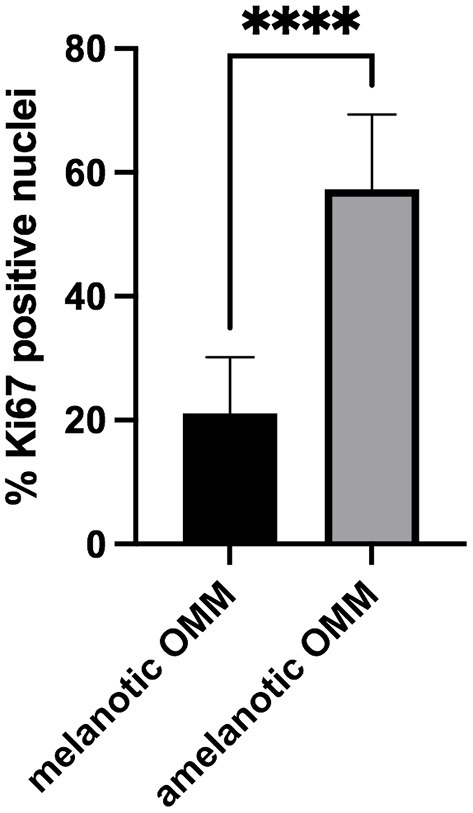
Scores of the Ki67-positive nuclei in samples of canine melanotic and amelanotic OMM. Means were compared using the Student's *t*-test. The percentage of Ki67-positive cells was significantly higher (****) in amelanotic OMM than melanotic OMM (*p* < 0.05).

The percentage of Ki67-positive nuclei was significantly higher in amelanotic OMM than in melanotic OMM, as shown in Table 3 and Figure 3.

### Correlation Between 5mC and Ki67 Scorings

The correlation between 5mC score in melanotic and/or amelanotic OMM and the Ki67 score was evaluated by the Spearman's correlation coefficient, a non-parametric measure of rank correlation. It assesses how well the relationship between two variables can be described using a monotonic function. This coefficient assumes values between−1 and 1. Table 4 shows the degrees of correlations obtained. Significant low to moderate correlations were obtained when the 5mC and Ki67 from 38 samples were tested. No significant correlations were observed when either melanotic or amelanotic 5mC scores were compared with the respective Ki67 score.

**Table 4:** Summary of the Spearman's correlation analysis of the 5mC data and Ki67 positive nuclei in melanotic and amelanotic OMM.

**Data used for correlation**	**Spearman r**	**Correlation**	***P*-value**	**Significant?**
**Melanotic** **+** **amelanotic OMM (** ***n=*** **38)** **×** **Ki67 (** ***n=*** **38)**				
5mC 2+ × Ki67	0.3166	Positive, low	0.0528	^*^
5mC 1+ × Ki67	0.4439	Positive, moderate	0.0052	^**^
5mC negative × Ki67	−0.4426	Negative, moderate	0.0054	^**^
**Melanotic OMM (*****n=*****19)×Ki67 (same samples**, ***n=*****19)**				
5mC 2+ × Ki67	0.4368	Positive, moderate	0.0615	ns
5mC 1+ × Ki67	0.04561	Positive, very low	0.8529	ns
5mC neg × Ki67	−0.02807	Negative, very low	0.9092	ns
**Amelanotic OMM (*****n=*****19)×Ki67 (same samples**, ***n=*****19)**				
5mC 2+ × Ki67	0.1255	Positive, low	0.6087	ns
5mC 1+ × Ki67	0.2386	Positive, low	0.3253	ns
5mC negative × Ki67	−0.1719	Negative, low	0.4815	ns

* and ** * = significance level, ns = non significant*.

### Quantification of the Global DNA Methylation in Canine Blood Leukocytes by HPLC

**Figure 4:**
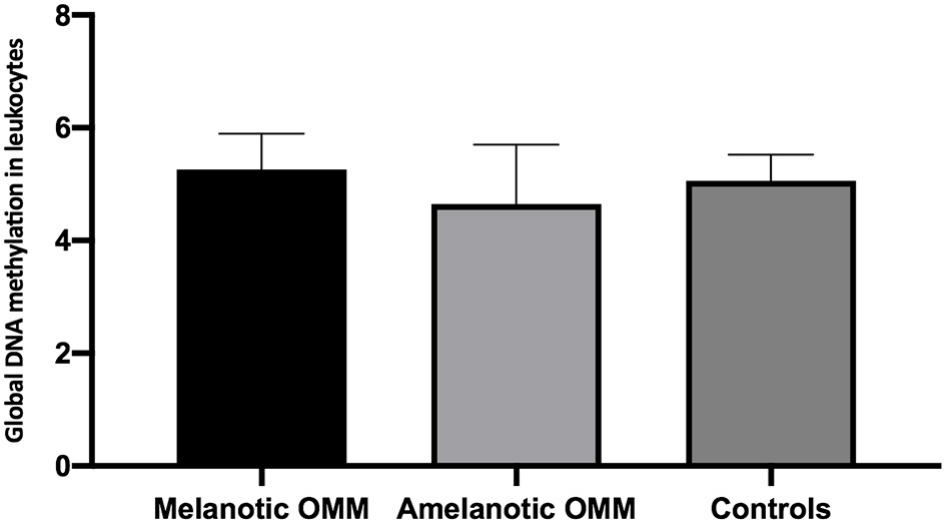
Quantification of global DNA methylation in peripheral blood leukocytes from dogs bearing melanotic OMM, amelanotic OMM, or control dogs. No statistical differences were obtained when the means were compared by ANOVA (*p* >0.05).

Quantification of global DNA methylation in leukocytes collected from melanotic OMM, amelanotic OMM, and control dogs was performed according to the area obtained in the chromatograms that refer to dC and 5mC. By analyzing the chromatograms, peripheral blood leukocytes from melanotic OMM and amelanotic OMM patients and controls were found to have a similar global DNA methylation content (5.26 ± 0.64, melanotic OMM; 4.64 ± 1.05, amelanotic OMM; and 5.06 ± 0.46, controls, *P-*value ≥0.05) (Figure 4).

**Figure 5:**
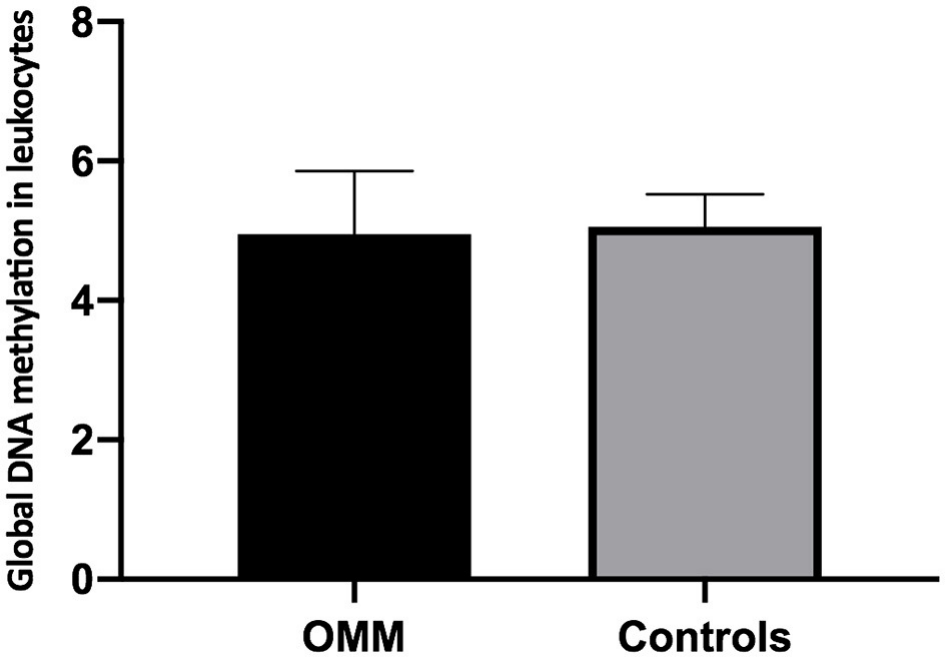
Quantification of global DNA methylation in peripheral blood leukocytes from dogs bearing melanotic and amelanotic OMM or control dogs. No statistical differences were obtained when means were compared by the Student's *t*-test (*p* > 0.05).

When leukocyte global DNA methylation in melanotic OMM and amelanotic OMM were combined and compared to the controls, no significant difference was obtained by the Student *t*-test (4.95 +0.90, OMM and 5.06 ± 0.46, controls) (*p* > 0.05) (Figure 5).

## Discussion

OMM is a very prevalent neoplasm in dogs. A report from our group have revealed differences in cell proliferation (mitosis countings), expression of connexins, and outcome between melanotic OMM and amelanotic OMM (6). Although several parameters have been identified to establish the behavior and prognosis of OMM (2, 26), only few studies have investigated the differences between melanotic and amelanotic OMM (6, 9, 28). To our knowledge, studies evaluating the global DNA methylation landscape in canine OMM and in blood leukocytes of OMM bearing dogs have not been reported in the literature, which motivated us to carry out the present study.

This study included OMM from both female and male dogs, aged 10–16 years old. Dogs with melanotic OMM, amelanotic OMM, and clinically healthy (blood leukocyte control) were enrolled in the cohort. OMM is reported to mainly affect animals aged 8–11 years (1) or middle-aged to older dogs, with average age being 9 years, and no gender predilections (21). This study included a homogeneous population of older dogs, and most of the dogs enrolled herein were females (52.64% females and 47.36% males).

Several histopathological parameters have been studied to predict the prognosis of OMM in dogs, including morphological classification, nuclear atypia, mitoses, cellular pleomorphism, margin evaluation, pigmentation, and cell proliferation (29). In the present study, tumor global DNA methylation and growth fraction were assessed to compare melanotic OMM to amelanotic OMM.

Initially melanotic OMM were differentiated from amelanotic OMM via gross observation of the tumor mass and morphological characteristics based on histopathological examination, as well as by estimating the number of cells that expressed melanin, according to the criteria of the Oncology Pathology Working Group OPWG (26). All amelanotic melanomas included in this study were whitish upon gross examination and had <50% of melanin-expressing cells based on the histopathological assessment. In addition, all amelanotic OMMs were subjected to immunohistochemistry with Melan A as the primary antibody, showing positivity. Such finding assured that amelanotic OMM was diagnosed with certainty.

Both genetic and epigenetic events are involved in carcinogenesis and cancer (14–16). Epigenetic events include DNA methylation, histone acetylation, and micro-RNA. Because epigenetic studies in canine melanomas are scarce, this study aimed to compare global DNA methylation in melanotic OMM to that in amelanotic OMM.

We performed immunohistochemical analysis of melanotic and amelanotic OMMs using 5mC as the primary antibody. All 38 samples, separated in 2 sets of 18 and 20 samples, respectively, were subjected to IHC in the same batch to avoid differences in DAB (for amelanotic OMM) or AEC (for melanotic OMM) revealing time as well the potential differential intensity that may be induced. The OMM slides were analyzed in an image analysis system to quantify strong, weak, or negative nuclei (at least 500 per case). The same quantification approach was used in other studies (18, 19, 30). The results of these quantifications showed that both melanotic and amelanotic OMMs presented higher percentages of weakly stained and negative 5mC nuclei. Such finding may presume global DNA hypomethylation in the OMM cells, according to the pioneer studies from Piyathilake et al. (17). The predominance of global DNA hypomethylation is in general associated with highly aggressive neoplasms. The negative nuclei have been interpreted as bearing a very low level of 5mC, which was impossible to detect using the immunohistochemical technique.

Although gene specific methylation was not evaluated, hypermethylation of cancer-related genes is known to play a fundamental role in tumorigenesis and may lead to increased invasion and possibly higher aggressiveness. Koroknai et al. (31) identified several methylation changes that can play a role in human melanoma progression, including hypermethylation of the promoter regions of the ARHGAP22 and NAV2 genes that are commonly altered in locally invasive primary melanomas as well as during metastasis. In addition, Yamamoto et al. (32) investigated the role of epigenetic aberrations in human melanomas by genome-wide DNA methylation analysis of 51 clinical malignant melanoma samples. Hierarchical clustering analysis revealed two DNA methylation epigenotypes: high- and low-methylation subgroups. Tumor thickness was significantly greater in cases of high-methylation tumors than low-methylation tumors. Further, the prognosis was significantly worse in high-methylation cases.

In the OMM samples, hypermethylated and hypomethylated nuclei were found, with higher numbers in hypomethylation cells. New studies isolating these cells may be performed to evaluate gene-specific DNA methylation in these cells.

In contrast (18, 19, 30), demonstrated that the most aggressive tumor subtypes have shown predominant weakly stained nuclei that presumably revealed global DNA hypomethylation. Unfortunately, in this study, we could not evaluate the normal methylation pattern of canine mucosal melanocytes, since these cells are roughly seen in histological sections of oral mucosa in clinically healthy dogs.

Many studies have revealed a decrease in general methylation with aging, and epidemiological analyses have used leukocyte methylation as a risk marker, suggesting a predisposition to cancer by DNA hypomethylation (21, 22). In our study, we investigated the level of global DNA methylation in aged dogs only, since the study protocol has not included the analysis of blood leukocytes of dogs from other ages. However, this can be approached in future studies.

Some studies have reported aberrant DNA methylation profiles in canine melanomas. By employing genome-wide next-generation sequencing to analyze the DNA methylation patterns of canine melanomas, studies found increased methylation at thousands of normally unmethylated CpG sites in CGIs and decreased methylation at normally methylated CpG sites in non-CGIs. Six genes with “sequence-specific DNA binding” annotation were significantly enriched, including three homeobox genes (HMX2, TLX2, and HOXA9). As a result, the researchers concluded that these genes may be involved in the tumorigenesis of canine malignant melanoma (33). Another recent study examined the DNA methylation status of long interspersed nucleotide element-1 (LINE-1) as a surrogate marker of genome-wide methylation changes in this disease. These studies compared 41 canine melanoma patient samples and six cell lines to normal mucosae. Based on their findings, LINE-1 showed remarkable hypomethylation in melanomas compared to normal mucosae (34).

Cell proliferation assessed via immunostaining and quantification of Ki67 can be considered an important biomarker of tumor aggressiveness and prognosis. Therefore, we sought to quantify the Ki67-positive nuclei in the same melanotic and amelanotic OMM samples according to the guidelines of Meuten et al. (29). Amelanotic OMM has a significantly higher Ki67 index than melanotic OMM; this is despite both tumors displaying Ki67 indexes higher than 19.5 cells/high power field (hpf), which indicates poor prognosis (29).

Smedley et al. (28) revealed that the value of the Ki67 index is useful as a prognostic factor among other histological features. Sevastre et al. (35) also analyzed cell proliferation (Ki67) in canine melanoma samples and found a positive correlation between Ki67 index value, necrosis, and mitosis. Therefore, by considering cell proliferation, we suggest that amelanotic OMM has a higher growth fraction (36) than melanotic OMM in dogs.

Interstingly, the 5mC scores in melanotic and amelanotic OMM revealed a low to moderate positive correlation with Ki67 scores, while the negative characteristic (absence of 5mC staining) was negatively and moderately correlated with the Ki67 score. This may indicate that if a neoplasm shows many negative 5mC nuclei, it may show a higher growth fraction. This may be in accordance with the presumed relationship between the global DNA hypomethylation and the higher cell proliferation and possibly higher aggressiveness.

Epigenetic alterations may be affected by environmental modifications. The assessment of global DNA methylation in circulating leukocytes is thus being carried out to understand the environmental influences on cancer (23, 24). The role of global DNA methylation in blood leukocytes is relevant to the determination of possible etiological factors and pathogenesis of melanoma; this is because it is affected by lifestyle and environmental factors and is potentially a molecular link mediating the association between unhealthy lifestyle/environmental carcinogens and cancer risk (23).

DNA methylation has been intensively investigated as an epigenetic factor with potential biomarkers for cancer and carcinogenesis. According to the results of this study, the pattern of circulating leukocyte global DNA methylation in dogs bearing melanotic and amelanotic OMM was similar based on the HPLC methods employed. However, a tendency for hypomethylation was noted in the amelanotic melanomas. Further, leukocyte global DNA methylation in combined melanotic and amelanotic OMMs did not differ from that observed in control animals. Such findings indicate that canine OMM may not be associated with environmental or systemic factors. Thus, local etiological factors may be considered. Local factors related to OMM pathogenesis may include consanguinity, trauma, chemical exposure, hormones, genetic susceptibility, the presence of pigmented cells, or even the oral microbiota. Inflammation may also be associated with the etiology of these tumors (2).

In conclusion, this study revealed the presence of higher 5mC weakly stained nuclei, presumably global DNA hypomethylation, in both canine melanotic and amelanotic OMM, and significantly higher growth fraction in amelanotic OMM than melanotic OMM detected by Ki67 scoring. No alterations were found when the leukocyte global DNA methylation was compared among melanotic, amelanotic, and healthy dogs of similar ages. The study's main limitation is its relatively low number of melanoma samples. However, to our knowledge, this is the first report to evaluate global DNA methylation in canine melanomas and peripheral blood leukocytes. Canine OMM is considered to be a good model for human OMM (3, 7–9, 37). Notably, in present day clinics, there has been translation of new cancer treatments from pet dogs to humans (38). Further, findings regarding tumors from one species may influence the prevention and treatment in another. As the differentiation between melanotic and amelanotic melanomas can be important to understanding the prognosis and new possibilities of intervention, a study involving a higher number of OMM cases is ongoing.

## Data Availability Statement

The raw data supporting the conclusions of this article will be made available by the authors, without undue reservation.

## Ethics Statement

The animal study was reviewed and approved by Committee of Ethics on the Use of Animals (CEUA) of the School of Veterinary Medicine and Animal Science (Process number: 9522230517) and the School of Medicine of the University of São Paulo (Process number: 997/18). Written informed consent was obtained from the owners for the participation of their animals in this study.

## Author Contributions

NS: conceptualization, methodology, conducted the study, and writing the manuscript. TE, FH-B, and AL: methodology and editing the manuscript. KC: supplied animal materials for the study and editing the manuscript. JF: methodology and data analysis. CM: supplied materials for the study, methodology, and editing the manuscript. MD: conceptualizadion, funds, writing the manuscript, supervision, and editing the manuscript. All authors contributed to the article and approved the submitted version.

## Conflict of Interest

The authors declare that the research was conducted in the absence of any commercial or financial relationships that could be construed as a potential conflict of interest.

## Publisher's Note

All claims expressed in this article are solely those of the authors and do not necessarily represent those of their affiliated organizations, or those of the publisher, the editors and the reviewers. Any product that may be evaluated in this article, or claim that may be made by its manufacturer, is not guaranteed or endorsed by the publisher.
